# Integrating Physiological Biomarkers into Precision Livestock Farming for Laying Hens: Methodological Challenges and Future Research Directions

**DOI:** 10.3390/ani16193155

**Published:** 2026-10-08

**Authors:** Leecheon Kim, Darae Kang

**Affiliations:** 1 Department of Animal Science, Jeonbuk National University, Jeonju 54896, Republic of Korea; rladlcjs4483@naver.com; 2 Department of Animal Biotechnology, Jeonbuk National University, Jeonju 54896, Republic of Korea

**Keywords:** laying hen, precision livestock farming, physiological biomarkers, machine learning, animal welfare

## Abstract

Continuous environmental, production, locational, image, and acoustic data have increasingly been collected from laying-hen farms through precision livestock farming (PLF) systems. However, sensor-derived classifications have often been validated against human-assigned behavioral or appearance labels rather than independently measured physiological states. In this narrative review, the methodological requirements for using physiological indicators as reference labels in PLF models for laying hens were examined, considering blood biochemistry, corticosterone concentration, the heterophil-to-lymphocyte ratio, body temperature, and immune or disease indicators alongside structured farm-level data and unstructured video, thermal, and acoustic data. Direct sensor-to-reference comparison studies numbered only seven and predominantly used rectal or cloacal temperature, peripheral temperature, clinical trajectories, or disease outcomes. No study identified in this review developed and externally validated a composite label combining blood biochemistry, the heterophil-to-lymphocyte ratio, corticosterone, and immune or pathogen indicators in laying hens. Major barriers were considered to include sensor–biomarker time-scale mismatches, sampling-induced label noise, and inconsistent units of analysis across individual, flock, and farm levels. It is suggested that future studies incorporate individual identification, biomarker-specific temporal alignment, multimodal composite labeling, and independent farm-level validation, so that PLF for laying hens can progress from sensor-centered detection toward biologically validated decision support linked to management actions.

## Simple Summary

Precision livestock farming (PLF) systems have been developed to continuously collect environmental, production, location, image, and acoustic data from laying-hen farms. However, the accurate detection of a behavioral or image-based pattern does not necessarily indicate that health or welfare status has been correctly assessed. In this review, the extent to which physiological indicators—including blood biochemistry, corticosterone, the heterophil-to-lymphocyte ratio, body temperature, and immune or disease measures—can serve as reference labels for validating PLF models was examined. Direct sensor-to-reference comparison studies in laying hens numbered only seven, with most relying on body temperature or clinical and disease outcomes alone. No study identified in this review was found to have developed and externally validated a composite label integrating blood biochemistry, stress, immune, and pathogen indicators simultaneously. To generate biologically meaningful decision support, it is suggested that future PLF studies (1) identify individual birds, (2) align sensor signals with biomarker-specific temporal windows, (3) combine complementary physiological measures, and (4) validate resulting models on independent farms.

## 1. Introduction

Recently, the laying hen industry has seen expanded adoption of automated systems that continuously collect environmental data such as temperature, humidity, and noxious gas concentrations, along with feed and water intake and egg production performance. In parallel, research applying machine learning to video, acoustic, and radio-frequency identification (RFID) data to detect behavioral and health abnormalities has also increased. The core value of precision livestock farming (PLF) lies not in the sensor values themselves, but in translating continuously observed animal responses into management decisions [1,2,3]. However, because commercial systems and much of the existing literature remain heavily weighted toward environmental and flock-level productivity indicators, high classification accuracy does not necessarily equate to a biologically valid assessment of welfare.

Animal welfare and health status constitute a multidimensional concept that must be assessed by considering behavior, morphology, productivity, and physiological responses [4]. Blood biochemistry, corticosterone (CORT), the heterophil-to-lymphocyte (H/L) ratio, and immune or pathogen indicators can serve as biological reference measures for interpreting sensor-derived changes [5,6]. In this review, a label denotes the reference outcome assigned to a sensor-data window for model development; a ground-truth label is appropriate only when its biological definition, sampling protocol, timing, and unit of analysis are explicit. A composite label is defined here as one that integrates at least two indicator classes (e.g., blood biochemistry, stress, and immune or pathogen measures) obtained from the same individuals within a specified time window, and external validation is defined as the evaluation of a trained model on data from at least one farm not included in model training; these definitions are applied consistently in Section 4 and Conclusions. These reference measures are intermittent and frequently invasive, whereas smart-farm data are collected continuously at high frequency. The key gap is therefore not simply the availability of physiological indicators, but the limited number of studies that align measurements with different temporal, individual, and spatial resolutions and then test whether the resulting relationship is reproducible on independent farms.

Accordingly, the objectives of this review were threefold: first, to compare the characteristics of physiological indicators and PLF sensor data; second, to identify the temporal, sample size, label, and unit-of-analysis issues that arise when physiological indicators are applied as ground-truth labels in artificial intelligence (AI) models; and third, to propose strategies involving non-invasive measurement, multimodal fusion, hierarchical modeling, and external validation. Whereas previous reviews of AI in the laying hen industry have primarily addressed technical advances in sensors and algorithms, the present review focuses on the extent to which sensor-derived predicted states validly reflect actual physiological health and welfare.

## 2. Materials and Methods

This narrative review was conducted to map and critically assess how sensor-derived measures in laying hens have been linked to physiological, clinical, or disease-related reference outcomes. The search was designed to address the methodological problem of label construction and validation in artificial intelligence enabled PLF; it was not conducted as a systematic review or meta-analysis.

Scopus and PubMed were searched on 20 September 2026 without a publication-year restriction. English-language records were retrieved using three concept-block searches, each combining terms for laying hens or pullets with two further term sets restricting to (i) PLF/sensor/AI technologies and physiological or biomarker terms, (ii) PLF/sensor/AI technologies and welfare, health, or behavioral terms, or (iii) physiological, biochemical, or immune terms and measurement-validation terms (e.g., validity, reliability, sampling, assay, circadian); the complete Boolean strings executed in each database are reported verbatim in Supplementary Table S1. The broad combined search included terms for PLF, sensors, accelerometry, radio-frequency identification, computer or thermal vision, acoustics, activity monitoring, machine or deep learning, physiology, biomarkers, corticosterone, the heterophil-to-lymphocyte ratio, biochemistry, body temperature, immune status, pathogens, clinical signs, and vaccination. The searches retrieved 2602 records (Scopus, 1835; PubMed, 767). After deduplication by DOI, PMID, Scopus EID, and exact title, 1735 unique records remained.

Studies were eligible if they involved laying hens or pullets (*Gallus gallus domesticus*); no restriction was placed on study design. Studies conducted in other poultry or livestock species were retained only where they addressed a methodological concept not yet established in laying hens (e.g., label validation or non-invasive stress assessment) and are identified as such in the text. The search was restricted to English-language title and abstract fields, which can still retrieve a source whose full text is not in English when an English title and abstract are indexed; one included study [7] is such a case, published in a Chinese-language journal with an English title and abstract, and this is noted where it is cited. The complete Boolean query strings executed in each database, together with their retrieved bibliographic records (title, authors, year, and identifier), are provided in Supplementary Table S1 to support the verification of the totals reported above. Consistent with the narrative, rather than systematic, design of this review, a stage-by-stage screening log (records screened at title/abstract versus assessed at full text) was not maintained. The three concept-block searches were run separately per database (PubMed: 81, 161, and 525 records; Scopus: 227, 441, and 1167 records, respectively); the largest search in each database (block iii) combined physiological, biochemical, or immune terms with measurement-validation terms rather than with PLF/sensor terms, which accounts for its greater size and for the presence of records unrelated to sensor-based monitoring among its results. Of the retrieved records, seven primary studies directly compared a sensor-derived measure against an independently measured physiological, clinical, or disease-related reference outcome in laying hens or pullets at the individual-bird level; these constitute the core evidence base for the evidence-of-absence claim addressed in Section 4 and the Conclusions. The overall literature identification and evidence-selection process is summarized in Supplementary Figure S1.

Titles and abstracts were screened for relevance to sensor-based monitoring in laying hens or pullets. Primary studies were prioritized when sensor-derived measures were compared with physiological, clinical, disease, or temperature-related reference outcomes. Studies addressing only production, environmental control, image-based detection, or behavioral classification without a relevant biological or clinical reference were used only as supporting context when they informed label construction, data integration, or model validation. Reviews, methodological papers, and authoritative guidance were used to contextualize physiological indicators and validation concepts. Reference lists and citation chaining were used to identify additional background literature.

## 3. Physiological Indicators and PLF Data Streams in Laying Hens

Accurate assessment of welfare and health status in laying hens requires confirmation of internal physiological changes that cannot be captured by morphological observation or productivity indicators alone [8]. These indicators can be broadly classified into blood biochemical indicators, stress indicators (hormones), and disease-related indicators, each of which is obtained through distinct sampling methods and analytical procedures (Table 1). A caveat common to all three categories is that none reflects a single controlled variable: each is jointly shaped by pre-analytical handling, individual biological state (age, laying stage, strain), and the sampling matrix and timing used, so that a value recorded at one time point rarely constitutes a sufficient label on its own [5,6,9]. This constraint is well established in the physiology literature and is not revisited for each indicator below; instead, the following subsections focus on what is methodologically distinct about each indicator category, and on what has—and has not—been achieved when these indicators are combined with sensor-derived proxies in practice.

Blood biochemical indicators provide complementary information on energy, lipid, and protein metabolism and on tissue injury. Glucose and cholesterol are commonly interpreted with energy and lipid status; total protein and albumin with nutritional and synthetic status; AST with injury involving the liver and skeletal muscle; and uric acid with avian nitrogen metabolism. Because ALT has limited hepatic specificity in birds, AST and ALT should be interpreted with other clinical, production, and laboratory data rather than as definitive stand-alone liver-function markers [10]. Reference [10] concerns companion avian species rather than laying hens; it is cited here for general avian clinical-chemistry interpretation and is extrapolated in the absence of a laying-hen-specific source for these ranges. Reference values also depend on age, laying stage, strain, feed intake, sampling time, and analytical method.

### 3.1. Stress and Endocrine Indicators

For AI-label construction, the most important issue is not whether CORT or the H/L ratio is a useful stress marker in general, but whether the measured value represents the biological state assigned to the sensor window. CORT can change during capture and sampling, whereas the H/L ratio reflects a response on a different time scale; the two measures should therefore not be treated as interchangeable labels [17,18,19].

A concrete example of integrated monitoring is a vaccine-challenge study in which wearable activity patterns were interpreted together with changes in cloacal temperature and clinical scores [20]. This design does not establish a blood-biomarker label, but it demonstrates how a sensor-derived trajectory can be evaluated against independently measured biological outcomes at the individual-bird level.

Pre-analytical metadata should be recorded together with each physiological result: individual identity, sampling time, capture-to-sampling interval, sample matrix, anticoagulant, hemolysis status, feeding condition, storage duration, and freeze–thaw history. These details permit later assessment of whether observed variation reflects biology or the measurement process [21,22].

Fecal CORT metabolites (FCM) allow for repeated, non-invasive sampling but are influenced by excretion delay, diurnal variation, diet, strain, and fecal output; the excretion rate should therefore be considered in addition to concentration [11]. Feather CORT may provide a cumulative signal over the feather-growth period, but feather site, growth stage, washing, extraction, and contamination must be standardized [12,13]. Blood, fecal, and feather CORT capture acute, sub-daily, and cumulative stress-response windows, respectively, and should not be used as interchangeable proxies.

### 3.2. Immune and Disease-Related Indicators

Non-invasive sampling does not by itself guarantee a valid label; when introducing a new sample matrix—for example, egg white (albumen) CORT, which may not agree with mass spectrometry results because of cross-reactivity in immunoassays [23]—accuracy, precision, limit of detection, recovery, repeatability, and parallelism (consistency of response across biological substrates) must each be validated [24,25]. Liquid chromatography–tandem mass spectrometry (LC-MS/MS) is recommended as the reference method against which such immunoassays should be validated.

Disease-related data include total and differential white blood cell counts, H/L ratio, serum antibody titers, inflammatory/acute-phase response indicators, and pathogen antigen or nucleic acid detection results [14,15,16]. Antibody titers reflect past exposure or vaccine response, and a positive PCR result indicates only nucleic acid detection and does not necessarily signify clinical disease or the presence of an infectious pathogen. Conversely, an inappropriate target sample or sampling time point may result in an infected individual being classified as negative. For AI training, the assay method, diagnostic criteria, sample type and collection time point, vaccination history, clinical findings, and the individual/flock unit of analysis must all be recorded together [26,27].

Analytical procedures vary widely, including blood testing, excreta examination, and pathological confirmation via necropsy, and the time required and accuracy differ depending on the method used.

The three physiological indicators described above share a common practical constraint: all require capturing the individual bird to obtain a sample. This characteristic stands in contrast to the real-time, non-invasive data collection methods used in smart farming, described in the following section, and provides the background for understanding the challenges of integrating these two data types.

Emerging molecular tools such as RT-qPCR, digital droplet PCR, multiplex pathogen panels, mucosal immunity transcriptomics, and acute-phase proteins (e.g., ovotransferrin, serum amyloid A) may further refine disease-related labels, but a systematic treatment of assay-level diagnostics is beyond the scope of this review.

PLF generates structured records (environment, production, and individual-access events) and unstructured records (video, thermal images, and sound). Table 2 and Table 3 summarize the available data types; the discussion below focuses on the properties that determine whether these records can be linked validly to physiological or clinical reference outcomes.

### 3.3. Structured Data: Environmental, Productivity, and Individual Identification Data

Environmental and production records are usually collected continuously, whereas their biological interpretation is often limited by their aggregation at house or flock level. Temperature, humidity, gas concentrations, feed and water intake, egg production, and mortality can describe exposure or population response, but they do not necessarily represent the exposure or state of an individual bird [28,29]. Calibration, missing-data handling, time synchronization, and location metadata are therefore required before these variables are used for model development (Table 2).

RFID and related identification systems can convert visits to feeders, drinkers, nest boxes, or functional zones into individual-level event records. These data enable repeated within-bird analysis, but read rate, signal interference, antenna placement, and the rule used to group raw reads into a visit must be validated because they directly affect the derived behavioral measure [30,31,32]. Similar RFID feeding/drinking validation concerns have been raised in broilers [31].

**Table 2:** Characteristics of structured data collected in laying hen smart farms.

Category	Representative Indicators	Collection Level	Collection Frequency	Quality Control	Modeling Considerations
Environment	Temperature, humidity, THI, CO_2_, NH_3_, light intensity, wind speed	Sensor location/house	Seconds–minutes	Calibration, drift, missing data, location metadata	House-level average values may not represent individual-level exposure
Production	Egg production, egg weight, feed/water intake, mortality	Cage/flock	Hours–days	Equipment error, manual data entry, inventory correction	May reflect outcome or lagged indicators rather than causal factors
Individual location and access logs (RFID/UWB)	Tag-read records of feeder/nest-box access (TagID, AntennaID, TimeStamp)	Individual	Seconds–minutes (event-based)	Tag loss/detachment (handled as missing data), variation in read rates across antenna locations, threshold settings for gap-filling	Different systems are used depending on flock size (LF RFID for small flocks, UHF RFID for large flocks); the threshold chosen to group read intervals into a single behavioral event can directly affect duration and frequency estimates; noise arises depending on tag position and orientation
Management events	Feeding, lighting, transfer, vaccination, treatment	House/flock	Event-based	Accurate timestamps and recorder identification	Should be included as confounder or intervention variables

### 3.4. Unstructured Data: Video, Thermal Imaging, and Acoustic

Video, thermal imaging, and acoustic sensing provide non-invasive proxies for activity, posture, feather condition, thermal state, and vocalization. Their value for this review lies not in automated detection alone, but in whether the derived proxy can be matched to an individual, a biologically plausible time window, and an independently measured physiological or clinical outcome [33,34].

Video-based tracking can produce individual trajectories and behavioral durations, but occlusion, overlap, illumination, and ID switching may introduce error before the data are linked to a biological label. RFID- or marker-based systems can assist individual identification, yet their accuracy must be assessed at the same level as the intended physiological comparison [32,35].

Computer-vision models can detect individuals and classify feather condition, comfort behavior, damaging pecking, or piling. CNNs learn image features directly from frames, whereas YOLO-family models detect objects and their locations in a single processing step. These approaches establish technical detection performance, but do not by themselves establish that the detected feature reflects physiological health or welfare [36,37,38].

Acoustic models can classify vocalization patterns and their associations with environmental conditions or performance, while multimodal models can combine video, acoustic, environmental, and physiological inputs. For either approach, the relevant standard is whether the sensor-derived signal is evaluated against a predefined reference outcome and then tested in temporally or geographically independent data [39,40] (Table 3).

**Table 3:** Applications and limitations of unstructured and multimodal data in laying hens.

Data Type	Extracted Features/Tasks	Example Applications in Laying Hens	Representative Models	Key Limitations	References
RGB video/imagery	Individual detection/tracking, posture, activity index	Dustbathing, degree of feather damage, piling, floor eggs, activity index/individual tracking	YOLO, CNN, tracking (DeepSORT, StrongSORT, ByteTrack)	Occlusion, lighting conditions, stocking density	[36,37,38,41,42,43]
Thermal imaging—plumage assessment	Quantitative assessment of feather coverage/damage severity, tracking of age-related feather loss progression	Plumage-coverage scoring, feather-damage quantification, age-related feather-loss tracking	Temperature-range thresholding/segmentation, ROC-analysis-based threshold setting	Influenced by ambient environment, distance, angle, and feather color	[44,45,46]
Thermal imaging—heat stress	Estimation of acute/chronic heat-stress intensity, use as a surrogate for core body temperature	Heat-stress severity classification, core-temperature surrogate estimation	Linear regression/mixed models, variance-component estimation (animal model), Random Forest	Sensitive to imaging distance and head angle; environmental temperature differences across seasons/production cycles; relatively lower performance at certain body regions such as legs, feet, and comb	[47,48,49,50]
Acoustic data	MFCC, frequency, vocalization events	Vocalization classification (by stress stimulus/age/exposure timing)	CNN, spectrogram	Overlapping vocalizations	[39,51]
Multimodal data	Integration of video + acoustic + environmental + physiological data	(Limited) integration of behavioral/environmental exposure with physiological labels	Early/intermediate/late fusion	Synchronization, missing modality, cost, external validation (quantified via domain transfer score)	[40]

Thus, PLF data are valuable because they are non-invasive and repeatedly measured, not because they intrinsically provide a welfare or health label. Their integration with physiological measures requires individual identification where possible, explicit temporal alignment, sensor quality control, and a reference outcome defined at the same level of analysis.

## 4. Current Evidence Linking Sensor Data to Physiological Status

Most laying-hen PLF studies remain proxy-oriented: RFID and video systems can quantify feeding, nest-box use, location, peaking, dustbathing, piling, and feather condition, but their outputs are usually evaluated against human observation, environmental conditions, or production data rather than against concurrently measured physiological or clinical outcomes. High agreement for a behavioral or image label therefore establishes technical detection performance, not physiological validity (Table 4).

Direct integration studies provide more informative examples. Solis et al. [50] used thermal-image features to classify heat-stress grades defined by rectal temperature, thereby linking a sensor signal to a reference measure with direct thermoregulatory relevance. Song et al. [7] (published in a Chinese-language journal with an English title and abstract) combined thermal images with environmental variables to predict temperature measured by a wearable sensor beneath the wing; this design illustrates multimodal prediction, but its reference was a local surface-adjacent temperature rather than rectal or implanted core temperature, and the reported R^2^ and RMSE (Table 4) were obtained by 5-fold internal cross-validation on six hens at a single farm, rather than by external validation. These studies show that thermal imaging can be biologically anchored, while also demonstrating why the anatomical location and definition of the reference measurement must be stated explicitly.

Other integrated designs demonstrate that the choice of outcome determines the strength of the resulting claim. Activity-sensor data have been assessed alongside physiological, clinical, and behavioral changes after a vaccine challenge [20], and RFID-derived patterns have been examined as predictors of subsequent health outcomes [54]. Thermal responses have also been associated with acute stress intensity [47]. Such studies move beyond behavior-only classification because the sensor output is evaluated against a biological or clinical outcome. Nevertheless, concurrent association is not equivalent to a transportable predictive model: temperature, age, strain, housing, and handling may influence both the sensor feature and the reference measure. Robust studies should therefore progress from synchronized repeated measurements to individual- or flock-level holdout validation, temporally separated testing, and independent-farm evaluation.

Accordingly, the current evidence supports a cautious conclusion. Sensor systems are promising tools for identifying biologically meaningful risk signals, but few laying-hen studies have yet demonstrated that a model trained with explicitly defined physiological or clinical labels remains calibrated and useful across new farms. This evidence gap, rather than sensor accuracy alone, should guide future study design and reporting.

Table 4 also reveals a critical evidence gap. Studies that directly anchored sensor-derived measures to biological or clinical outcomes predominantly used rectal or cloacal temperature, peripheral temperature, clinical trajectories, or disease outcomes. No study identified in this review developed and externally validated a model using a composite label that combined blood biochemistry, the H/L ratio, corticosterone, and immune or pathogen indicators in laying hens. This absence should be interpreted as a priority for future research, rather than as evidence that such physiological labels are unnecessary. Some sensor-versus-parasite studies were not included in Table 4 under this criterion; for example, Willems et al. [56] linked night-time activity patterns to poultry red mite infestation, but infestation was quantified only at the cage level rather than for individual birds.

## 5. Methodological Barriers to Label Construction and Validation

The use of physiological indicators as ground-truth labels for sensor-based models is constrained by three interrelated methodological barriers, addressed in turn below.

### 5.1. Mismatch in Time Scale and Lag

Smart-farm sensors provide real-time input at the scale of seconds or minutes, whereas physiological indicators require anywhere from several hours to several days from sample collection to analysis. This makes it unclear which point in the sensor data stream should be matched to an indicator value obtained at a given time, and renders alignment of the two data types on a common time axis difficult. This time lag can substantially reduce the accuracy of training data, particularly for physiological states that change rapidly, such as stress or disease.

### 5.2. Small Sample Sizes and Mismatch in Unit of Analysis

Invasive sampling induces considerable stress during capture and blood collection, and animal welfare principles require that the number of animals used and repeated sampling procedures be minimized and refined [57,58]. This fundamentally constrains the acquisition of the large-scale datasets required by deep learning models. In practice, blood is often sampled from only a small subset of individuals within an entire flock. When individual-level labels are then assigned to whole-flock video footage or house-average sensor values, this mismatch in the unit of analysis can give rise to ecological fallacy and pseudo-replication [59,60,61].

### 5.3. Label Noise and Uncertainty in Case Definition

In machine learning model training, physiological indicators are typically used as ground-truth labels; however, for blood-based stress indicators, the very process of capture and blood collection can induce acute stress that distorts the measured value, making the reliability of the label a critical barrier. This means that the label itself already contains noise, and no matter how sophisticated the algorithm applied, this fundamental limitation cannot be fully overcome.

The three problems described above (time lag, limited sample size, and label noise) do not arise independently, but instead derive from a single underlying cause: the invasive nature of physiological indicators. Therefore, addressing these problems requires the joint pursuit of technical approaches that reduce invasiveness itself and algorithmic approaches that make effective use of limited data; the following section examines specific strategies for overcoming these challenges.

## 6. Model Validation and Reporting

AI research on laying hens using physiological labels must be validated by distinguishing among biological validity, which evaluates the association between the proxy and the physiological state; analytical validity, which evaluates assay-level performance (e.g., limit of detection, analytical sensitivity and specificity, intra- and inter-assay repeatability, recovery, and parallelism); diagnostic validity, which evaluates how accurately the sensor-derived classification identifies the clinical or physiological condition of interest (diagnostic sensitivity and specificity, and positive and negative predictive values, which vary with field prevalence); and external validity, which evaluates performance across new strains, ages, housing facilities, and seasons and should be demonstrated through spatial (cross-farm), temporal (across production cycles), and management-level holdout schemes [20,62]. The unit of study and the level of the label must be matched, and data splitting must ensure that data from the same individual or flock are not simultaneously included in both the training and test sets. Model performance should preferably be reported together with measures of uncertainty, calibration, handling of missing data, false alarm rate, and the thresholds linked to management intervention. Reporting should follow the STROBE-Vet statement for observational studies generating baseline reference data, together with STARD-AI for diagnostic-accuracy studies and TRIPOD+AI for prediction-model studies, as applicable [26,27,63]. Only when such a validation framework is in place can it be determined whether high predictive performance observed on internal data is maintained on new farms and translates into actual improvements in welfare.

**Figure 1:**
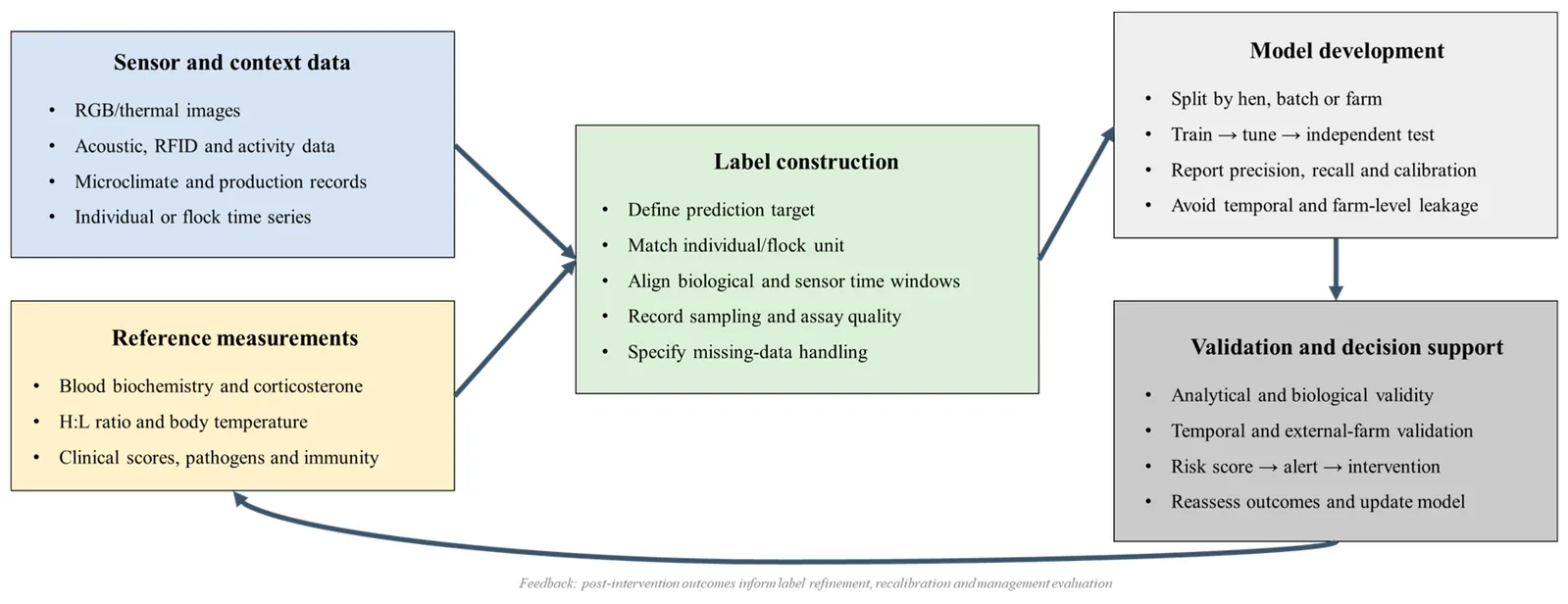
Framework for biologically validated sensor-based monitoring in laying hens. Sensor and context data (including RFID, radio-frequency identification) are integrated with reference measurements during label construction; model development is followed by analytical, biological, temporal, and external validation; post-intervention outcomes are then fed back to inform label refinement, recalibration, and management re-evaluation, closing the cycle. Arrows between boxes indicate the direction of the workflow, and arrows within boxes indicate the order of steps. H:L, heterophil-to-lymphocyte; RGB, red–green–blue.

Sensor input, physiological ground truth, data integration, model validation, and management application should be linked in a cyclical manner (Figure 1). Imaging, thermal, acoustic, RFID, environmental, and production data are integrated following individual identification, temporal alignment, and quality control, and are subsequently validated against blood biochemistry, CORT concentration, the H/L ratio, body temperature, and immune and pathogen-related indicators. Rather than serving as a final determinant, model output should be presented as a risk score with associated uncertainty that can be reviewed and acted upon by the manager, and the outcomes of such interventions should, in turn, be incorporated into model re-evaluation and subsequent training.

Figure 1 illustrates the conceptual pipeline rather than a matrix-specific protocol; the sample matrix, sensor modality, and reference method associated with each indicator are detailed in Table 1 and Table 4.

## 7. Integrative Strategies and Future Research Directions

The issues of temporal lag, limited sample size, and label noise discussed above can be mitigated through improvements in data acquisition methods as well as algorithmic compensation. These two aspects are addressed separately below.

### 7.1. Non-Invasive Surrogate Matrices

To address the label noise problem, that is, the distortion of measured values caused by handling stress during sample collection, research on non-invasive surrogate indicators has been actively pursued. For stress hormones, metabolites accumulated in feathers or excreta can be measured instead of blood, thereby excluding the confounding effect of acute stress induced by the sampling procedure itself [11,12]. For disease-related indicators, early anomaly detection studies using imaging-based posture analysis have been conducted in broilers to capture abnormal signs prior to the onset of clinical symptoms [64], and attempts to detect equipment malfunction or abnormal behavior at an early stage using real-time changes in activity level and spatial distribution patterns have likewise been reported in broiler houses [65]; whether these approaches transfer to laying hens, whose housing and behavior differ substantially from broilers, has not been established.

### 7.2. Sensor-Based Surrogate Indicators

A second approach is to acquire sensing-based surrogate indicators that indirectly estimate physiological status from imaging and acoustic sensors. Image-based behavioral analysis primarily estimates early signs of stress or disease through gait, activity level, and grooming patterns, whereas acoustic sensors are used to detect abnormalities in respiratory sounds or vocalization patterns for the early detection of respiratory disease.

Thermal imaging represents a well-documented example linking physiological reference values with sensor signals. Facial surface temperature in laying hens responds to environmental conditions, although the response varies by anatomical region and its relationship with the temperature–humidity index (THI) has not been consistent [49]; robustness to thermal environment differs among strains [66], and also varies depending on the mode of exposure, such that a rapid rise in THI can exert a more severe effect on mortality than a gradual increase [67,68]. In a meta-analysis, strain, age, and stocking density interacted significantly with heat sensitivity, whereas the daily amplitude of temperature variation alone did not exert a significant effect on most traits [67].

That is, the rate of temperature rise and its duration appear to be more closely related to the actual hazard than the amplitude of temperature fluctuation itself. Surface temperature has been associated with core body temperature and thermoregulatory status in pullets [52] and separately in broiler chickens [69]; this association has not been established specifically in adult laying hens, and acute stress itself also alters skin temperature [47]. Recently, a study classifying thermal imaging features in laying hens using rectal temperature as the label has been reported [50], although validation designs that avoid treating repeated measurements from the same individual at the same time point as independent samples remain necessary.

Since these image- and acoustic-based proxies do not directly measure physiological status, criterion validity should be established through concurrent measurement with laboratory indicators, repeated measurements should be taken while accounting for the temporal precedence between sensor signals and physiological indicators, and predictive performance and calibration should be verified in independent farms.

In particular, infrared thermography has been shown to correlate significantly with feather score through mean image temperature or the area proportion within a specific temperature range [44]; however, because it is influenced by ambient temperature, humidity, airflow, imaging distance, and feather condition, it should not be regarded as a direct measure of body temperature or stress [45,48]. Indeed, in the ROC analysis of [46], sensitivity and specificity for discriminating severe plumage damage (score 0 vs. 2) exceeded 95% for the back and belly plumage in both white- and brown-feathered hens, whereas sensitivity for discriminating intact from early or moderate damage (score 0 vs. 1) was considerably lower across all three body regions, ranging from 31.7% to 71.5%. This indicates that thermography accurately captures damage that has already progressed but carries a substantial risk of missing subtle changes at an early stage, underscoring its limitations as a physiological early-warning indicator.

### 7.3. Data Alignment Reflecting Biological Time Windows

Temporal alignment should reflect the response, excretion, and accumulation time windows of physiological indicators, rather than simply matching a single sensor value to the sampling time point. Plasma CORT reflects an acute response occurring on the order of minutes, FCM reflect a delayed response associated with gastrointestinal transit, and feather CORT reflects a cumulative signal integrated over the growth period; applying the same fixed window to all labels therefore risks training the model to treat biologically distinct events as equivalent outcomes. The window length and lag should be predefined, and a sensitivity analysis should be performed accordingly.

This temporal-axis mismatch can be addressed by grouping sensor data within a defined interval before and after the sampling time point into a single window, or by interpolating values between sampling points to estimate a continuous signal, thereby allowing two datasets with different temporal resolutions to be handled on a common time-series axis. Where sensor and label sequences are both time-ordered but not evenly synchronized, elastic alignment methods such as Dynamic Time Warping offer a further option for matching corresponding segments across the two series.

### 7.4. Small-Sample Learning and Independent Validation

Reviews of machine learning in agriculture and livestock have emphasized that data quality and independent validation are as important as algorithm selection [70], and CNN-based livestock imaging studies have repeatedly reported limitations arising from restricted farm numbers and imaging conditions, imbalanced labels, and insufficient external validation [71]. In particular, because consecutive frames captured at high frame rates exhibit very high correlation between adjacent frames, data splitting should be performed at the level of date, individual, flock, or farm rather than at the frame level.

For the small-sample problem, transfer learning, self-supervised learning, data augmentation, and few-shot learning can be applied [72,73].

Data augmentation increases the number of frames or windows but not the number of independent biological replicates; images or windows from the same individual should therefore remain within the same data split. For imbalanced outcomes, sensitivity (the proportion of true cases detected), specificity (the proportion of non-cases correctly rejected), precision (the proportion of model-positive predictions that are true), recall (sensitivity), and F1-score should be reported alongside accuracy. The area under the precision-recall curve (AUPRC) is generally more informative than the area under the receiver operating characteristic curve (AUROC) when positive cases are uncommon [74]. Calibration, which assesses agreement between predicted and observed risk, should be reported with discrimination metrics [63]. Final evaluation should focus on predictive performance and false-alarm rate in new flocks, farms, and seasons rather than internal cross-validation alone.

The countermeasures discussed above correspond respectively to the three problems raised earlier. Improvements in data acquisition (non-invasive surrogate indicators and sensing-based surrogate indicators) fundamentally mitigate the problems of label noise and limited sample size, whereas the algorithmic approaches (time-series alignment and small-sample compensation) represent a complementary strategy that maximizes the utility of already-acquired data. These two approaches are not mutually exclusive, and it is considered desirable that they be applied in parallel in actual practice.

## 8. Mid-to-Long-Term Research Directions

Whereas the countermeasures presented above have focused primarily on the form of data and the analytical techniques themselves, the following two topics may be regarded as mid- to long-term research directions that address, at a more fundamental level, inter-individual heterogeneity and the structural limitations of data accumulation.

### 8.1. Individual Baselines and Hierarchical Modeling

Because physiological indicators have different baselines across individuals, models based on absolute values risk mistaking inter-individual variation for an anomalous signal, or conversely misjudging a genuine anomaly as falling within the normal range; an approach that focuses on relative change from each individual’s baseline is therefore needed.

Statistically, mixed-effects models or hierarchical Bayesian models can account for the nested structure of individual birds within cages, flocks, and farms. Hierarchical Bayesian models estimate these levels jointly and quantify uncertainty in each level-specific effect. In machine-learning analyses, individual-specific baseline normalization or learning of within-individual change can serve a similar purpose [56,75]. However, simply including individual ID as an input variable does not generalize to unobserved birds and can cause information leakage; validation should therefore hold out new individuals as well as, where possible, new flocks or farms [76,77].

### 8.2. Data Standardization and Inter-Institutional Collaboration

Because the ethical and cost constraints associated with invasive sampling impose a fundamental limit on the sample size and number of repeated measurements attainable by any single research institution, the long-term establishment of public databases or consortia integrating physiological data across multiple institutions is warranted. Such infrastructure could achieve sample sizes unattainable by individual studies, thereby improving the generalization performance of deep learning models.

However, sharing of raw data across institutions is often impractical in reality due to concerns regarding personal data protection, proprietary information, and data ownership. As an alternative, federated learning, in which each institution trains a model locally without releasing raw data and shares only the resulting model parameters for aggregation, has drawn attention as a feasible approach that simultaneously satisfies the competing demands of data security and large-scale training [78,79]. Nevertheless, heterogeneity in data distribution across farms, communication cost, model convergence, and the potential for information leakage through model updates remain challenges to be resolved [80].

Beyond these technical and data-governance barriers, commercial deployment of biomarker-validated PLF systems remains constrained by practical and economic factors: the labor and veterinary time required for invasive sampling, the cost of assays run at a scale sufficient for model training and periodic recalibration, and the data-infrastructure investment (connectivity, storage, and integration across sensor types) needed to sustain continuous monitoring on commercial farms. These constraints do not diminish the biological case for physiological labels, but they mean that non-invasive and sensor-based surrogate indicators (Section 7) are likely to remain the more immediately deployable path for most producers, with invasive reference measures reserved for periodic validation and model recalibration rather than routine use.

Although operating at different levels, correction for individual variation (at the modeling level) and the establishment of data-sharing infrastructure (at the data-ecosystem level) share the common goal of generalizing knowledge derived from data limited to a small number of individuals or institutions to a broader scope. As empirical validation accumulates along these two directions in the future, the integration of physiological indicators with smart-farm data is expected to be realized at a more substantive level.

## 9. Conclusions

This review analyzed the requirements for integrating physiological indicators as ground-truth labels for AI models in precision poultry farming for laying hens. Current technology can detect behavior, appearance, location, vocalization, and environmental changes with high accuracy; however, whether such detection performance actually reflects physiological health and welfare has not been sufficiently validated. In particular, indirect proxy studies relying on human-assigned behavioral and appearance labels have predominated over studies involving direct integration of physiological indicators.

The core problem limiting the utilization of physiological labels is not merely low measurement frequency. Rather, differing response times and accumulation periods across indicators, measurement error induced by capture and blood sampling, unit-of-analysis error arising from assigning values obtained from a limited number of individuals to the entire flock, information leakage from repeated-measures data, and domain shift across farms operate simultaneously. Individual identification, indicator-specific time windows, hierarchical data structure, and external farm validation should therefore be designed prospectively.

Future AI systems for laying hens should transition from a stage focused on improving the classification accuracy of a single sensor toward biologically validated multimodal decision-support systems. Physiological indicators operating on different time scales should be integrated as complementary composite labels rather than as interchangeable substitutes, and calibration, uncertainty, and false-alarm rate of predicted values, together with outcomes following management intervention, should be evaluated collectively.

This review did not perform a quantitative meta-analysis, and for some methodologies, evidence from other poultry species or livestock was used as supplementary support due to a paucity of studies conducted directly in laying hens; this constitutes a limitation. Nevertheless, by presenting physiological validity, data alignment, unit of analysis, and external validation within a single framework, this review has specified priorities for future research design and data standardization.

## Figures and Tables

**Table 1:** Measurement characteristics of physiological indicators in laying hens and considerations for their application as AI labels.

Category	Representative Indicators	Major Physiological Significance	Major Confounding Factors	Sampling/Measurement	Considerations for AI Labels	References
Blood biochemistry	Glucose, cholesterol, total protein, albumin	Reflects energy, lipid, and protein metabolism, as well as nutritional status	Age, laying stage, feeding, time to blood collection, hemolysis, analyzer	Serum/plasma biochemical analysis	Rather than relying on a single population-level reference range, within-individual variation and assay-specific reference ranges should be used	[10]
Tissue damage/nitrogen metabolism	AST, ALT, CK, GLDH, uric acid	Provides auxiliary assessment of hepatic and muscular damage and of nitrogen excretion	Lack of tissue specificity, exercise/capture stress, dietary protein content, dehydration	Serum/plasma biochemical analysis	Hepatic function should not be assessed on the basis of AST/ALT alone	[10]
Stress	Plasma corticosterone, H/L ratio	Reflects acute HPA-axis activation as well as relatively sustained hematological responses	Duration of capture/handling, circadian rhythm, repeated exposure, infection/inflammation	Blood immunoassay/blood smear	Because the two indicators operate on different measurement timescales, interpretation should integrate multiple markers	[5,6]
Non-invasive stress	Fecal corticosterone metabolites (FCM), feather corticosterone	Represents a cumulative signal of hormone metabolism—including excretory lag—or of hormone deposition accumulated over the feather growth period	Diet, strain/line, fecal volume, site of feather growth, external contamination	Immunoassay following collection of fecal or feather samples	Not a direct or immediate substitute for plasma values; matrix-specific validation is required	[11,12,13]
Immunity/disease	Leukocyte count, H/L ratio, antibody titer, PCR/antigen detection	Reflects host response, vaccination or exposure history, or detection of pathogen-specific targets	Vaccination history, type of target sample, stage of infection, assay sensitivity/specificity	Blood, smear, fecal, or environmental sample analysis	A positive result does not equate to clinical disease; case definitions and diagnostic criteria must be explicitly stated	[14,15,16]

**Table 4:** Core studies linking sensor-derived measures with physiological, clinical, or disease-reference outcomes in laying hens and pullets. The table includes studies in which a sensor-derived measure was evaluated against an independently measured physiological, clinical, or disease outcome at the individual-bird level; studies in which the reference outcome was assessed only at the group or cage level were treated as supporting context in the main text rather than as core studies in this table.

Study	Population/Setting	Sensor-Derived Measure	Reference Measurement or Outcome	Biological Matrix/Reference Method	Main Finding	Critical Limitation
[50]	192 hens from two strains; climate-controlled heat-stress and comfort chambers	Infrared thermal signatures from featherless regions (face, eye, wattle, comb, leg, and foot)	Rectal temperature; danger versus normal heat-stress class	Core body (rectal); digital thermometry	Random Forest classification AUC: 89.0% (face) and 88.3% (wattle); accuracy: 83.4% (face) and 83.6% (wattle); the two metrics rank the regions in opposite order	Controlled chambers; temperature-threshold classification; no external validation
[20]	20 laying hens; 5 batches; live ILT vaccine challenge (day 6) after a saline control (day 3)	FitBark activity level and distance travelled; TrackLab distance travelled	Total clinical score (five clinical signs) and cloacal temperature	Core body (cloacal) and ordinal clinical scoring	Activity and distance travelled declined significantly with day (*p* < 0.001) and were highly significantly associated with total clinical scores (*p* < 0.001)	Association design, not a predictive model; activity had already declined after the saline control, before vaccination; small sample (*n* = 20), single facility, no external validation
[7]	Commercial caged farm; 121 five-minute observation windows	Head infrared temperature corrected with NIR/depth images, plus environmental variables	Wearable under-wing temperature	Under-wing skin-adjacent; wearable temperature sensor (not a laboratory assay)	Random Forest: R^2^ = 0.696; RMSE = 0.246 °C (5-fold internal cross-validation; 6 hens, single farm)	Under-wing temperature is a surrogate rather than direct core temperature; one farm; no external validation
[52]	648 pullets; four thermal treatments	Infrared temperature at eight body regions	Cloacal temperature	Core body (cloacal); digital thermometry	Strong region-specific associations; regression prediction errors below 0.4 °C for several regions	Controlled pullet study; applicability to adult commercial hens remains untested
[53]	Indoor-reared laying hens exposed to enrichment withdrawal and intermittent handling	Infrared temperature of comb, face, and eye	Plasma corticosterone and behavioural markers	Blood plasma; corticosterone immunoassay	Surface-temperature trajectories reflected chronic and intermittent stress exposure	Not a predictive model; the magnitude and direction of change are context-dependent
[54]	Free-range laying hens; individual health outcomes at the end of production	RFID range-use features with machine learning	Spotty liver disease, roundworm, and tapeworm outcomes	Whole-bird post-mortem; necropsy and parasitological examination	Correctly predicted 28% of spotty liver disease, 33% of roundworm, and 33% of tapeworm outcomes	Highly imbalanced outcomes and modest predictive performance
[55]	Five free-range laying hens monitored for six days	Harness accelerometer-derived locomotor activity	Subcutaneous biologger body temperature	Subcutaneous (implanted); biologger telemetry (not a laboratory assay)	Locomotor activity and body temperature showed a significant 24-h circadian association	Very small sample; invasive implanted logger; not a disease-prediction study

## Data Availability

No new data were created or analyzed in this study. Data sharing is not applicable to this article. The literature search records compiled during the review process are provided in Supplementary Table S1.

## Supplementary Materials

The following supporting information can be downloaded at: https://www.mdpi.com/article/10.3390/ani16193155/s1, Figure S1: Literature identification and evidence selection flow for this narrative review; Table S1: Complete Boolean search strings and bibliographic records for each database search.

## Abbreviations

The following abbreviations are used in this manuscript:

PLF	Precision livestock farming
RFID	Radio-frequency identification
CORT	Corticosterone
H/L	Heterophil-to-lymphocyte
AI	Artificial intelligence
FCM	Fecal corticosterone metabolites
THI	Temperature–humidity index

## References

[1] Berckmans D. (2017). General introduction to precision livestock farming. Anim. Front..

[2] Norton T., Chen C., Larsen M.L.V., Berckmans D. (2019). Precision livestock farming: Building ‘digital representations’ to bring the animals closer to the farmer. Animal.

[3] Okinda C., Nyalala I., Korohou T., Wang J., Achieng T., Wamalwa P., Mang T., Shen M. (2020). A review on computer vision systems in monitoring of poultry: A welfare perspective. Artif. Intell. Agric..

[4] Dabbou S., Mugnai C., Soglia D., Tarantola M., Macchi E., Sartore S., Bergagna S., Pagliasso G., Schiavone A. (2022). Multifactorial evaluation of regrouping effects on performance and welfare in two Italian dual-purpose chicken breeds: Bianca Di Saluzzo and Bionda Piemontese. Animals.

[5] Mumma J.O., Thaxton J., Vizzier-Thaxton Y., Dodson W. (2006). Physiological stress in laying hens. Poult. Sci..

[6] Shini S., Shini A., Huff G. (2009). Effects of chronic and repeated corticosterone administration in rearing chickens on physiology, the onset of lay and egg production of hens. Physiol. Behav..

[7] Song D., Luo S., Zhu Y., Tong Q., Wang H., Wang L. (2025). Temperature measurement method for commercially farmed layer hens based on multi-source image and environmental data fusion. Trans. Chin. Soc. Agric. Mach..

[8] Nielsen S.S., Alvarez J., Bicout D.J., Calistri P., Canali E., Drewe J.A., Garin-Bastuji B., Gonzales Rojas J.L., Gortázar Schmidt C., EFSA Panel on Animal Health and Welfare (AHAW) (2023). Welfare of laying hens on farm. EFSA J..

[9] Alm M., Tauson R., Holm L., Wichman A., Kalliokoski O., Wall H. (2016). Welfare indicators in laying hens in relation to nest exclusion. Poult. Sci..

[10] Harr K.E. (2002). Clinical chemistry of companion avian species: A review. Vet. Clin. Pathol..

[11] Alm M., Holm L., Tauson R., Wall H. (2014). Corticosterone metabolites in laying hen droppings—Effects of fiber enrichment, genotype, and daily variations. Poult. Sci..

[12] Häffelin K., Lindenwald R., Kaufmann F., Döhring S., Spindler B., Preisinger R., Rautenschlein S., Kemper N., Andersson R. (2020). Corticosterone in feathers of laying hens: An assay validation for evidence-based assessment of animal welfare. Poult. Sci..

[13] Bartels T., Berk J., Cramer K., Kanitz E., Otten W. (2021). Research Note: It’s not just stress—Fecal contamination of plumage may affect feather corticosterone concentration. Poult. Sci..

[14] Buharideen S.M., Hassan M.S., Najimudeen S.M., Niu D., Czub M., Gomis S., Abdul-Careem M.F. (2021). Immune responses in laying hens after an infectious bronchitis vaccination of pullets: A comparison of two vaccination strategies. Vaccines.

[15] Lee C., Kim J.H., Kil D.Y. (2022). Comparison of stress biomarkers in laying hens raised under a long-term multiple stress condition. Poult. Sci..

[16] Onizawa E., Westman M.E., Bogema D.R., Deutscher A.T., Eamens K., Micallef M.L., McDonogh T., Jenkins C. (2025). Application of a Quantitative Real-Time PCR Assay for Early Detection of Salmonella enterica Serovar Enteritidis on Poultry Farms During an Outbreak in New South Wales, Australia (2018–2020). Transbound. Emerg. Dis..

[17] Romero L.M., Romero R.C. (2002). Corticosterone responses in wild birds: The importance of rapid initial sampling. Condor.

[18] Scanes C.G. (2016). Biology of stress in poultry with emphasis on glucocorticoids and the heterophil to lymphocyte ratio. Poult. Sci..

[19] Weimer S.L., Wideman R.F., Scanes C.G., Mauromoustakos A., Christensen K.D., Vizzier-Thaxton Y. (2018). An evaluation of methods for measuring stress in broiler chickens. Poult. Sci..

[20] Kang H., Brocklehurst S., Haskell M., Jarvis S., Sandilands V. (2025). Do Activity Sensors Identify Physiological, Clinical and Behavioural Changes in Laying Hens Exposed to a Vaccine Challenge?. Animals.

[21] Owen J.C. (2011). Collecting, processing, and storing avian blood: A review. J. Field Ornithol..

[22] Wachholz L., Genova J.L., Polese C., Broch J., Savaris V.D.L., Eyng C., Junior N.R., Tsutsumi C.Y., Nunes R.V. (2023). Light intensity, blood fraction, fasting and storage time affect blood biochemical metabolites in broiler chickens. Semin. Cienc. Agrar..

[23] Caulfield M.P., Padula M.P. (2020). HPLC MS-MS analysis shows measurement of corticosterone in egg albumen is not a valid indicator of chicken welfare. Animals.

[24] Andreasson U., Perret-Liaudet A., van Waalwijk van Doorn L.J., Blennow K., Chiasserini D., Engelborghs S., Fladby T., Genc S., Kruse N., Kuiperij H.B. (2015). A practical guide to immunoassay method validation. Front. Neurol..

[25] U.S. Food and Drug Administration (FDA) (2018). Bioanalytical Method Validation: Guidance for Industry.

[26] Sargeant J., O’Connor A.M., Dohoo I., Erb H., Cevallos M., Egger M., Ersbøll A., Martin S., Nielsen L.R., Pearl D. (2016). Methods and processes of developing the Strengthening the Reporting of Observational Studies in Epidemiology–Veterinary (STROBE-Vet) statement. J. Vet. Intern. Med..

[27] Sounderajah V., Guni A., Liu X., Collins G.S., Karthikesalingam A., Markar S.R., Golub R.M., Denniston A.K., Shetty S., Moher D. (2025). The STARD-AI reporting guideline for diagnostic accuracy studies using artificial intelligence. Nat. Med..

[28] Astill J., Dara R.A., Fraser E.D., Roberts B., Sharif S. (2020). Smart poultry management: Smart sensors, big data, and the internet of things. Comput. Electron. Agric..

[29] Olejnik K., Popiela E., Opaliński S. (2022). Emerging precision management methods in poultry sector. Agriculture.

[30] Nakarmi A.D., Tang L., Xin H. (2014). Automated tracking and behavior quantification of laying hens using 3D computer vision and radio frequency identification technologies. Trans. ASABE.

[31] Li G., Zhao Y., Hailey R., Zhang N., Liang Y., Purswell J. (2019). An ultra-high frequency radio frequency identification system for studying individual feeding and drinking behaviors of group-housed broilers. Animal.

[32] Alindekon S., Rodenburg T.B., Langbein J., Puppe B., Wilmsmeier O., Wille S., Louton H. (2025). Development and computer-assisted validation of a radio frequency identification system for tracking individual chicken visits to functional areas. Poult. Sci..

[33] Li N., Ren Z., Li D., Zeng L. (2020). Automated techniques for monitoring the behaviour and welfare of broilers and laying hens: Towards the goal of precision livestock farming. Animal.

[34] Manikandan V., Neethirajan S. (2025). Ai-powered vocalization analysis in poultry: Systematic review of health, behavior, and welfare monitoring. Sensors.

[35] Nidhi M., Liu K., Flay K. (2025). Multiobject tracking in livestock—From farm animal management to state-of-the-art methods. Animal.

[36] Lamping C., Derks M., Koerkamp P.G., Kootstra G. (2022). ChickenNet—An end-to-end approach for plumage condition assessment of laying hens in commercial farms using computer vision. Comput. Electron. Agric..

[37] Sozzi M., Pillan G., Ciarelli C., Marinello F., Pirrone F., Bordignon F., Bordignon A., Xiccato G., Trocino A. (2023). Measuring comfort behaviours in laying hens using deep-learning tools. Animals.

[38] Bist R.B., Subedi S., Yang X., Chai L. (2023). A novel YOLOv6 object detector for monitoring piling behavior of cage-free laying hens. AgriEngineering.

[39] Lin X., Zhu W., Liu L., Zhou Z. (2025). Decoding Laying Hen Behavior and Physiological Status through Acoustic Biomarkers: Temporal Patterns, Rooster-Hen Vocalization Identification in Group Housing, and Environmental Adaptation. Poult. Sci..

[40] Essien D., Neethirajan S. (2025). Multimodal AI systems for enhanced laying hen welfare assessment and productivity optimization. Smart Agric. Technol..

[41] Subedi S., Bist R., Yang X., Chai L. (2023). Tracking floor eggs with machine vision in cage-free hen houses. Poult. Sci..

[42] Paneru B., Bist R., Yang X., Chai L. (2024). Tracking dustbathing behavior of cage-free laying hens with machine vision technologies. Poult. Sci..

[43] Yang X., Bist R., Paneru B., Chai L. (2024). Monitoring activity index and behaviors of cage-free hens with advanced deep learning technologies. Poult. Sci..

[44] Cook N., Smykot A., Holm D., Fasenko G., Church J. (2006). Assessing feather cover of laying hens by infrared thermography. J. Appl. Poult. Res..

[45] Zhao Y., Xin H., Dong B. (2013). Use of infrared thermography to assess laying-hen feather coverage. Poult. Sci..

[46] Schreiter R., Freick M. (2022). Is infrared thermography an appropriate method for early detection and objective quantification of plumage damage in white and brown feathered laying hens?. Poult. Sci..

[47] Herborn K.A., Graves J.L., Jerem P., Evans N.P., Nager R., McCafferty D.J., McKeegan D.E. (2015). Skin temperature reveals the intensity of acute stress. Physiol. Behav..

[48] Loyau T., Zerjal T., Rodenburg T., Fablet J., Tixier-Boichard M., Pinard-Van Der Laan M., Mignon-Grasteau S. (2016). Heritability of body surface temperature in hens estimated by infrared thermography at normal or hot temperatures and genetic correlations with egg and feather quality. Animal.

[49] Kim N.Y., Kim S.J., Oh M., Jang S.Y., Moon S.H. (2021). Changes in facial surface temperature of laying hens under different thermal conditions. Anim. Biosci..

[50] Solis I.L., de Oliveira-Boreli F.P., de Sousa R.V., Martello L.S., Pereira D.F. (2024). Using thermal signature to evaluate heat stress levels in laying hens with a machine-learning-based classifier. Animals.

[51] Neethirajan S. (2025). Decoding vocal indicators of stress in laying hens: A CNN-MFCC deep learning framework. Smart Agric. Technol..

[52] Cândido M., Tinôco I., Albino L., Freitas L., Santos T., Cecon P., Gates R. (2020). Effects of heat stress on pullet cloacal and body temperature. Poult. Sci..

[53] Herborn K.A., Jerem P., Nager R.G., McKeegan D.E.F., McCafferty D.J. (2018). Surface temperature elevated by chronic and intermittent stress. Physiol. Behav..

[54] Welch M., Sibanda T.Z., De Souza Vilela J., Kolakshyapati M., Schneider D., Ruhnke I. (2023). An initial study on the use of machine learning and radio frequency identification data for predicting health outcomes in free-range laying hens. Animals.

[55] Abecia J.A., Canto F., Plaza J., Nieto J., Palacios C. (2025). Circadian rhythm of locomotor activity and body temperature in free-range laying hens as measured by triaxial accelerometers and subcutaneous biologgers. J. Appl. Poult. Res..

[56] Willems S., Nijs H., Sleeckx N., Norton T. (2025). Monitoring Night-Time Activity Patterns of Laying Hens in Response to Poultry Red Mite Infestations Using Night-Vision Cameras. Animals.

[57] Tannenbaum J., Bennett B.T. (2015). Russell and Burch’s 3Rs then and now: The need for clarity in definition and purpose. J. Am. Assoc. Lab. Anim. Sci..

[58] Sneddon L.U., Halsey L.G., Bury N.R. (2017). Considering aspects of the 3Rs principles within experimental animal biology. J. Exp. Biol..

[59] Hurlbert S.H. (1984). Pseudoreplication and the design of ecological field experiments. Ecol. Monogr..

[60] Robinson W.S. (1950). Ecological correlations and the behavior of individuals. Am. Sociol. Rev..

[61] Lazic S.E., Clarke-Williams C.J., Munafò M.R. (2018). What exactly is ‘N’ in cell culture and animal experiments?. PLoS Biol..

[62] Gómez Y., Stygar A.H., Boumans I.J., Bokkers E.A., Pedersen L.J., Niemi J.K., Pastell M., Manteca X., Llonch P. (2021). A systematic review on validated precision livestock farming technologies for pig production and its potential to assess animal welfare. Front. Vet. Sci..

[63] Collins G.S., Moons K.G., Dhiman P., Riley R.D., Beam A.L., Van Calster B., Ghassemi M., Liu X., Reitsma J.B., Van Smeden M. (2024). TRIPOD+ AI statement: Updated guidance for reporting clinical prediction models that use regression or machine learning methods. BMJ.

[64] Zhuang X., Bi M., Guo J., Wu S., Zhang T. (2018). Development of an early warning algorithm to detect sick broilers. Comput. Electron. Agric..

[65] Kashiha M., Pluk A., Bahr C., Vranken E., Berckmans D. (2013). Development of an early warning system for a broiler house using computer vision. Biosyst. Eng..

[66] Mack L., Felver-Gant J., Dennis R., Cheng H. (2013). Genetic variations alter production and behavioral responses following heat stress in 2 strains of laying hens. Poult. Sci..

[67] Mignon-Grasteau S., Moreri U., Narcy A., Rousseau X., Rodenburg T., Tixier-Boichard M., Zerjal T. (2015). Robustness to chronic heat stress in laying hens: A meta-analysis. Poult. Sci..

[68] Kang S., Kim D.-H., Lee S., Lee T., Lee K.-W., Chang H.-H., Moon B., Ayasan T., Choi Y.-H. (2020). An acute, rather than progressive, increase in temperature-humidity index has severe effects on mortality in laying hens. Front. Vet. Sci..

[69] Giloh M., Shinder D., Yahav S. (2012). Skin surface temperature of broiler chickens is correlated to body core temperature and is indicative of their thermoregulatory status. Poult. Sci..

[70] Liakos K.G., Busato P., Moshou D., Pearson S., Bochtis D. (2018). Machine learning in agriculture: A review. Sensors.

[71] Li G., Huang Y., Chen Z., Chesser G.D., Purswell J.L., Linhoss J., Zhao Y. (2021). Practices and applications of convolutional neural network-based computer vision systems in animal farming: A review. Sensors.

[72] Jing L., Tian Y. (2020). Self-supervised visual feature learning with deep neural networks: A survey. IEEE Trans. Pattern Anal. Mach. Intell..

[73] Rather I.H., Kumar S., Gandomi A.H. (2024). Breaking the data barrier: A review of deep learning techniques for democratizing AI with small datasets. Artif. Intell. Rev..

[74] Saito T., Rehmsmeier M. (2015). The precision-recall plot is more informative than the ROC plot when evaluating binary classifiers on imbalanced datasets. PLoS ONE.

[75] Carslake C., Occhiuto F., Vázquez-Diosdado J.A., Kaler J. (2022). Repeatability and predictability of calf feeding behaviors—Quantifying between- and within-individual variation for precision livestock farming. Front. Vet. Sci..

[76] Coelho Ribeiro L.A., Bresolin T., Rosa G.J.d.M., Rume Casagrande D., Danes M.d.A.C., Dórea J.R.R. (2021). Disentangling data dependency using cross-validation strategies to evaluate prediction quality of cattle grazing activities using machine learning algorithms and wearable sensor data. J. Anim. Sci..

[77] Wang J., Yu Z., Chebel R.C., Yu H. (2025). Impact of cross-validation designs on cattle behavior prediction using machine learning and deep learning models with tri-axial accelerometer data. Smart Agric. Technol..

[78] Mao A., Huang E., Gan H., Liu K. (2022). FedAAR: A novel federated learning framework for animal activity recognition with wearable sensors. Animals.

[79] Arshad J., Irtisam A., Arif T., Rasheed M.S., Chauhdary S.T., Rahmani M.K.I., Almajalid R. (2024). A federated learning model for intelligent cattle health monitoring system using body area sensors and IoT. Egypt. Inform. J..

[80] Ganesan L.P., Krishnan S. (2025). Blockchain-enabled, nature-inspired federated learning for cattle health monitoring. Informatics.

