# Supplementary material for: Integrating Physiological Biomarkers into Precision Livestock Farming for Laying Hens: Methodological Challenges and Future Research Directions
Source: Animals (Basel). 2026 Oct 8;16(19):3155. doi: 10.3390/ani16193155 (PMC13663413; doi:10.3390/ani16193155)
Supplement: Supplementary file 1 [file animals-16-03155-s001.zip › animals-4612037-supplementary.pdf]

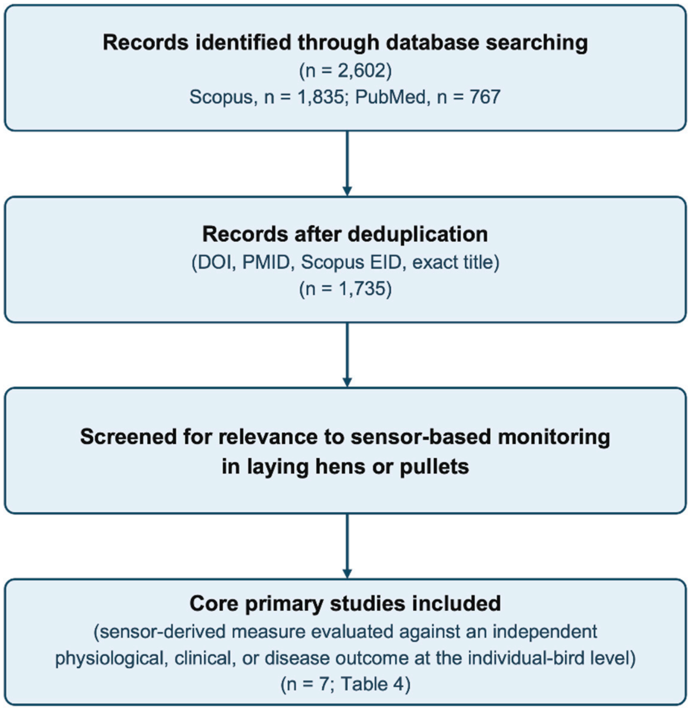

FigureS1. Literature identification and evidence selection flow

TableS1. Complete Boolean search strings and bibliographic records for each database search

Database	Search #	Concept-block combination	Complete Boolean query string (verbatim, as executed)	Records retriev	Search date	Source file
Scopus	1	Laying hen/pullet terms AND PLF/sensor/AI terms AND physiological/biomarker terms	("laying hen*" OR "layer hen*" OR "laying chicken*" OR pullet*) AND ("precision livestock farming" OR sensor* OR acceleromet* OR RFID OR "radio frequency identification" OR "computer vision" OR "machine vision" OR "thermal imaging" OR thermograph* OR acoustic* OR vocali* OR "activity monitor*" OR "machine learning" OR "deep learning") AND (physiolog* OR biomarker* OR corticosterone OR heterophil* OR lymphocyte* OR biochem* OR "body temperature" OR "core temperature" OR "cloacal [illegible]	227	2026-09-20	Scopus_Search1_2026-09-20_227.csv
Scopus	2	Laying hen/pullet terms AND PLF/sensor/AI terms AND welfare/health/behavior terms	TITLE-ABS-KEY (("laying hen*" OR "layer hen*" OR "laying chicken*" OR pullet*) AND ("precision livestock farming" OR sensor* OR acceleromet* OR RFID OR "radio frequency identification" OR "computer vision" OR "machine vision" OR "thermal imaging" OR thermograph* OR acoustic* OR vocali* OR "activity monitor*" OR "machine learning" OR "deep learning") AND (welfare OR wellbeing OR "well-being" OR health OR stress* OR disease* OR sickness OR behavior* OR behaviour*))	441	2026-09-20	Scopus_Search2_2026-09-20_441.csv
Scopus	3	Laying hen/pullet terms AND physiological/immune terms AND measurement-validation terms	TITLE-ABS-KEY (("laying hen*" OR "layer hen*" OR "laying chicken*" OR pullet*) AND (corticosterone OR glucocorticoid* OR heterophil* OR lymphocyte* OR "blood biochem*" OR "blood parameter*" OR "blood metabolite*" OR "biochemical parameter*" OR "body temperature" OR "core temperature" OR "cloacal temperature" OR "rectal temperature" OR "immune response*" OR "immune indicator*" OR "immune function" OR immunoglobulin* OR cytokine*) AND (validat* OR reliab* OR repeatab* OR reproducib* OR [illegible]	1167	2026-09-20	Scopus_Search3_2026-09-20_1167.csv
PubMed	1	Laying hen/pullet terms AND PLF/sensor/AI terms AND physiological/biomarker terms	("laying hen"[Title/Abstract] OR "laying hens"[Title/Abstract] OR "layer hen"[Title/Abstract] OR "layer hens"[Title/Abstract] OR "laying chicken"[Title/Abstract] OR "laying chickens"[Title/Abstract] OR pullet[Title/Abstract] OR pullets[Title/Abstract]) AND ("precision livestock farming"[Title/Abstract] OR sensor*[Title/Abstract] OR acceleromet*[Title/Abstract] OR RFID[Title/Abstract] OR "radio frequency identification"[Title/Abstract] OR "computer vision"[Title/Abstract] OR "machine vision"[Title/Abstract] OR [illegible]	81	2026-09-20	Pubmed_Search1_2026-09-20_81.txt
PubMed	2	Laying hen/pullet terms AND PLF/sensor/AI terms AND welfare/health/behavior terms	("laying hen"[Title/Abstract] OR "laying hens"[Title/Abstract] OR "layer hen"[Title/Abstract] OR "layer hens"[Title/Abstract] OR "laying chicken"[Title/Abstract] OR "laying chickens"[Title/Abstract] OR pullet[Title/Abstract] OR pullets[Title/Abstract]) AND ("precision livestock farming"[Title/Abstract] OR sensor*[Title/Abstract] OR acceleromet*[Title/Abstract] OR RFID[Title/Abstract] OR "radio frequency identification"[Title/Abstract] OR "computer vision"[Title/Abstract] OR "machine vision"[Title/Abstract] OR [illegible]	161	2026-09-20	Pubmed_Search2_2026-09-20_161.txt
PubMed	3	Laying hen/pullet terms AND physiological/immune terms AND measurement-validation terms	("laying hen"[Title/Abstract] OR "laying hens"[Title/Abstract] OR "layer hen"[Title/Abstract] OR "layer hens"[Title/Abstract] OR "laying chicken"[Title/Abstract] OR "laying chickens"[Title/Abstract] OR pullet[Title/Abstract] OR pullets[Title/Abstract]) AND (corticosterone[Title/Abstract] OR glucocorticoid*[Title/Abstract] OR heterophil*[Title/Abstract] OR lymphocyte*[Title/Abstract] OR "blood biochem*"[Title/Abstract] OR "blood parameter*"[Title/Abstract] OR "blood metabolite*"[Title/Abstract] OR [illegible]	525	2026-09-20	Pubmed_Search3_2026-09-20_525.txt
